# Congestive Malarial Fever

**Published:** 1873-05

**Authors:** Benjamin Rhett

**Affiliations:** South Carolina


### CONGESTIVE MALARIAL FEVER.

BY BENJAMIN RHETT, M.D., OF SOUTH CAROLINA.

The extreme fatality attendant upon the congestive and hemorrhagic forms of malarial fever renders desirable any additional light that can be thrown upon their successful treatment.

Having, after years of experience, gradually adopted a line of practice in the treatment of the severer types of malarial fever—(which practice, as far as my investigations into the views of treatment advised in standard works extend, differs materially from that accepted as the correct course)—I propose giving a sketch of my treatment, and my reasons therefor, accompanying the same with a relation of a few cases as illustrations.

This disease is known in some places as "congestive chill"—in other portions of the country as "congestive fever." The agent is evidently the malarial or paludal poison that causes bilious, remittent and intermittent fevers. The severity of the attack is dependent upon the virulence of the septic agent, the amount of the dose, or the susceptibility of the individual.

The first impression of the poisonous agent is evidently made upon the nervous filaments of the vagus and ganglionic nerves, distributed to the stomach and lungs—as the furred tongue and general *malaise* that precede attacks of malarial fever would indicate; the subsequent chill and constriction of the capillaries point to these parts of the nervous system as first implicated and impressed by the nocuous agent. The impression so made is evidently of an irritant and depressing nature.

These observations apply equally to all forms of malarial fevers dependent upon paludal poison.

In all pyrexias dependent upon the introduction of septic agencies from without, there are certain forms known a *fulminant*, owing to their rapid and explosive character, dependent, as above stated, upon the concentration of the poison, the amount absorbed, or individual susceptibility. Congestive chill and fever is the fulminant form of paludal fevers.

According to my observation, the third chill is most frequently the dangerous one; the two preceding attacks being often so mild and transitory as to render the patient and his friends uncertain as to the nature of the illness. Sometimes, however, the two preceding chills and febrile heats are well developed, and occasionally the congestive attack occurs in the course of chills and fever of some duration. The patient about the hour of, or a little before the hour of the previous day's attack, complains of drowsiness, chilliness, and general discomfort, and soon lapses into complete coma or semicomatose condition, a stupor from which he is with difficulty aroused; or he may be smitten so suddenly that he drops from an erect or sitting position, and is sometimes convulsed. I have known patients lie down to sleep, and the first intimation their friends would have of their illness would be the difficulty of arousing them.

The surface sometimes remains throughout the attack pale, cool, and bathed in perspiration; in others, the warmth is restored, but the perspiration remains; the skin of the hands and feet become shriveled and wrinkled, and the nails are blue; the face, and especially the nose and lips, purple and congested; the pupils of the eyes often contracted, and insensible to variations of light. The pulse increases in frequency, and is labored and oppressed.

Having found quinine potent as a febrifuge in bilious, remittent and intermittent fevers, I fully expected to experience benefit from it in the the treatment of the congestive forms of fever, especially as it had been so lauded in the Southwest when given in sixty and hundred-grain doses in the treatment of the malignant paludal fevers that scourged that section many years ago. But my hopes were disappointed. I gave it with alteratives (so-called); I gave it with alcoholic stimulants; and finally, I gave it with morphia, and then, for the first time, I observed beneficial results. I then suspected that the morphia was the active ingredient, and the quinia inert. I then experimented in ordinary attacks of chill and fever, and found that morphia had a decided effect in aborting or shortening the chill, and that quinia had no effect unless given during the intermission or remission of fever. I took a step yet further. I concluded that the reason of the quinia proving inert during the attack was, that being a bulky article, it required greater vitality in the digestive apparatus to take it up than they possessed, in their depressed and torpid condition; and that a more concentrated and active agent to allay nervous irritation, and one easy of absorption, must be used to arouse the nerves involved to healthy action, and that such an agent was morphia. Taking this view, and experimenting carefully with the class of remedies which were well known to combine diffusible properties and control over nervous irritation with concentration in bulk, I finally settled upon morphia, chloroform and camphor, and have been in the habit of administering them separately and in combination during the constrictive or chill stage, and during the congestive or reactive stage, and pushing these remedies to sufficient extent to secure an undoubted impression upon the disease. In addition to these remedies administered by mouth, I make use of turpentine enemata, and flannels wrung out with hot water and sprinkled with turpentine wrapped around the abdomen and back; and this use of turpentine is made not only for its derivative action, but for its well known control over capillary circulation—the capillaries of the external surface and internal organs being specially involved by the paralyzing influence of the poison, producing a stasis in, and distention of, these channels of circulation.

It is claimed for this method of treatment that it not only allays the depressing irritation caused by the poison of malaria, but that, by direct stimulation of the nervous system, it hastens the return of healthy action.

That I may be the better understood, we will draw a comparison between the effect of these remedies and quinia administered during the chill and febrile attack of ordinary intermittent or remittent fever. In most cases of fever, intermittent or remittent, twenty grains of quinia, administered during the intermission or remission, are sufficient to arrest the next paroxysm. But the same quantity, twenty grains, administered during a paroxysm, has no control over that paroxysm, either during the cold or hot stage, but exercises its influence, if it has any effect at all, on the succeeding paroxysm. Indeed, the febrile symptoms are aggravated by giving quinia during the paroxysm.

Suppose, now, we administer during the paroxysm, either during the cold or hot stage: ℞. Morphia, gr. ¼; chloroform, teaspoonful; camphor, gr. ii., and repeat, if necessary—the effect of the remedy is not only to *abort the chill,* but to *shorten the febrile paroxysm* by several hours. The same remedy, administered previous to the hour of chill, will sometimes postpone the attack several hours, and sometimes prevent it altogether.

I do not mean to supersede the use of quinia in treatment of these fevers, but to supplement its use by remedies that are invaluable when quinia is of little or no avail. In the intervals between the febrile paroxysm, quinia is undoubtedly *the drug* to prevent the incursion of the disease, but is of little or no use during the attack; and it is during the attack that the above concentrated "nervines" are of incalculable value.

The chief objection to their use is the rather obstinate vomiting they produce in some patients; but I have never failed to check this disagreeable symptom with effervescing draft (citric acid, bicarb. soda, or potassa).

Upon one occasion, mistaking the inception of an attack of typhoid fever, attended with congestion and delirium, I administered these remedies, and actually postponed the onset of the disease, or rather the development of fever, for a couple of days; but would certainly not recommend the experiment to be repeated where typhoid was diagnosed.

But I should certainly give these remedies a trial in hemorrhagic malarial fever, in some cases of which quinia exerts little control, and the continuance of febrile action produces rapid blood deterioration by loading the circulation with effete matter from tissue metamorphoses. But since adopting this treatment, I have not had a case of hemorrhagic fever to treat. But I know, to my sorrow, that quinia exercises little control over this form of fever—not that I think that any remedy is of much avail after the blood has become so deteriorated as to escape through the coats of the vessels, unless antiscorbutic remedies, combined with above concentrated stimulants, might possibly be used, as a dernier resort.

Case I.—Visiting, on the 12th of July, 1872, the wife of Mr. W. R., planter, he mentioned that he had had something like chills on that and the day previous. He was advised to take twenty grains of quinia next morning, some hours previous to the expected chill. He, on the morning of the 13th of July, after taking only a portion of the quinia, lay down to sleep, and slept to all appearance until two or three o'clock, at which time he was discovered to be in a stupor, when his family attempted to awake him. I arrived about five o'clock in the afternoon, and found him in the following condition: His surface pale, with cool extremities and cold nose; features pinched, and finger-nails blue; the pupils of his eyes contracted to the size of a pin's head, and little sensible to variations of light. Soon, the surface grew warmer, but the face, especially the nose and lips, became purple with venous congestion; the surface was soon bathed in profuse perspiration; the skin of the hands and feet became shriveled, the nails being of a purple color—the semi-comatose condition continuing. His breathing was embarrassed and stertorous, and there was considerable difficulty in swallowing. When partially roused to take medicine, his face had a wild, stupid glare, and he would lapse again into stupor with the spoon in his mouth. Having neither chloroform nor camphor with me, I administered one-fourth of a grain of morphia and twenty drops of turpentine every hour, plunged his feet into warm water, placed sinapisms upon legs, thighs and arms, and hot flannels, with turpentine, around the abdomen, and gave turpentine enemata. When chloroform and camphor were obtained, he took, every two hours—morphia, gr. $\frac{1}{4}$; chloroform, teaspoonful; camphor, gr. ii. This treatment was persevered in from five o'clock in the afternoon of the 13th of July to seven o'clock A.M. of the 14th, when there was a return to consciousness—a subsidence of stupor. A flow of urine, which had been suppressed, took place, and a subsidence of fever occurred. Quinia was given in full doses, and the patient soon rallied.

Case II.—I was called in August, 1872, to a negro boy, Dave Lee, about twelve years of age. He had been seized the day before with a congestive chill, following upon two mild attacks upon previous days. His case much resembled the foregoing, except that there was more delirium and less perspiration. The same treatment as in the last case was adopted, but in diminished doses adapted to his age. He died on the fourth day of his illness.

Case III.—Amanda Fair, a negro girl about thirteen years old, had been ailing for several days. Her sister returned from the bushes, whither Amanda and herself had gone, and reported that Amanda had been taken suddenly sick, and had fallen down. She was carried to the house, and I saw her about two hours after the inception of her attack. She had just come out of a convulsion when I arrived. Her case resembled the two foregoing, except that there was considerable delirium, great irritability of stomach, rendering it difficult to get the remedies retained, and that she had a couple of convulsions during the attack.

The same treatment was pursued. On the morning of the third day, consciousness returned, the fever subsided, and quinia in full doses was given. Although the most obvious action of remedies of this class—morphia, chloroform and camphor—is upon the cerebro-spinal system, yet we know that, either directly or through sympathy, that the nerves of organic life are also under their control. It is with the view that the stress of the poison falls upon the ganglionic system of nerves, and that these remedies quiet and subdue the depressing irritant action, and stimulate to healthy reaction, and their bulk, and capacity of absorption and diffusion, render them peculiarly appropriate in treatment of this disease, that they were adopted.

It is acknowledged that the practice is heroic. None other is of avail in so rapidly fatal a disease. A piddling or expectant practice is a waste of time. Dr. Lionel Beale says that the day of "expectant medicine" is past. The remark certainly holds good with regard to congestive chill and fever. Nature unaided, or without active, timely and efficient aid, is unable to cope with so powerful an enemy. Death is the almost certain lot of persons attacked by congestive chill and fever, unless relieved by active medical interference. Quinia in my hands, in large or small doses, alone or conjoined with stimulants, has failed to accomplish what I expected of it. I can confidently recommend the above class of remedies as every way more efficient in the treatment of malignant congestive fever during the paroxysm.

